# Biosynthetic Origin of the Methoxy Group in Quinine and Related Alkaloids

**DOI:** 10.1002/anie.202418306

**Published:** 2024-11-22

**Authors:** Blaise Kimbadi Lombe, Tingan Zhou, Lorenzo Caputi, Kerstin Ploss, Sarah E. O’Connor

**Affiliations:** Department of Natural Product Biosynthesis Max Planck Institute for Chemical Ecology Hans-Knöll-Straße 8, 07745 Jena, Germany

**Keywords:** Quinine, Cinchona, alkaloids, biosynthesis, genes discovery

## Abstract

Quinine is a historically important natural product containing a methoxy group that has been assumed to be incorporated at a late pathway stage. Here we show that the methoxy group in quinine and related alkaloids is introduced onto the starting substrate tryptamine. Feeding studies definitively show that 5-methoxytryptamine is utilized as a quinine biosynthetic intermediate *in planta*. We discover the biosynthetic genes that encode the responsible oxidase and methyltransferase, and we use these genes to reconstitute the early steps of the alkaloid biosynthetic pathway in *Nicotiana benthamiana* to produce a mixture of methoxylated and non-methoxylated alkaloid intermediates. Importantly, we show that the co-occurrence of both tryptamine and 5-methoxytryptamine substrates, along with the substrate promiscuity of downstream pathway enzymes, enable parallel formation of both methoxylated and non-methoxylated alkaloids.

Quinine (**1**), used for centuries as an antimalarial drug and as a bittering ingredient, is a member of the Cinchona alkaloids, a structurally diverse group of alkaloids produced primarily by *Cinchona* plants.^[1]^ In addition to quinine (**1**), the quinine stereoisomer, quinidine (**2**), is commonly prescribed as an antiarrhythmic medication.^[2]^ Non-methoxylated analogs (e.g. cinchonidine **3** and cinchonine **4**) also have established pharmacological properties^[1d,3]^ and, moreover, are used in synthetic chemistry as chiral catalysts and additives.^[4]^ Congeners with a single bond at C-10–C-11 (dihydro alkaloids; for structures, see **5**–**8** in Scheme S1 or Figure S1) also occur in several *Cinchona* species and have similar properties as **1**–**4**.^[1d,3a]^ While total syntheses of many Cinchona alkaloids have been reported,^[5]^ the biosynthesis of these compounds is still largely unelucidated.

Early investigations established that Cinchona alkaloids belong to the large family of monoterpenoid indole alkaloids.^[6]^ These alkaloids are derived from strictosidine (**12**), which is formed from the Pictet–Spengler condensation of a tryptophan-derived moiety, tryptamine (**10**), and the iridoid-type monoterpene secologanin (**11**).^[7]^ It has long been hypothesized that strictosidine is converted to cinchonidinone (**15a**) and cinchoninone (**15b**), compounds that are then methoxylated to form quinine (**1**) and quinidine (**2**), respectively (Scheme 1).^[8]^ Here we show through feeding experiments that methoxylation instead occurs on the early precursor tryptamine (**10**). We show that both tryptamine and 5-methoxytryptamine (**21**) are carried through the downstream pathway, leading to mixtures of methoxy (e.g. **1**, **2**) and non-methoxy (e.g. **3**, **4**) Cinchona alkaloids *in planta*. Additionally, we report the discovery of the biosynthetic genes that are responsible for this methoxylation, allowing reconstruction of the early biosynthetic steps of non-methoxy and methoxy Cinchona alkaloid intermediates.

Methoxylation of cinchonidinone (**15a**) and cinchoninone (**15b**) was first proposed^[8a]^ based on the incorporation of the radio-labeled compounds corynantheal (**13**) and cinchonidinone (**15a**) into quinine (**1**), though the incorporation was marginal (ca. 0.002%).^[8b,c]^ Additionally, methoxylated ketone **17a** has been detected in *C. ledgeriana*,^[8c]^ and crude extracts of a cell culture derived from this species showed the presence of NADPH-dependent reduction activity that specifically reduced **17 a/b** to **1** and **2**.^[8f]^ Moreover, a recently discovered *O*-methyltransferase from *C. pubescens* can methylate 6’-hydroxycinchoninone (**16a/16b**).^[8d]^ Together, these data are consistent with—though do not prove—a pathway order of hydroxylation of **15 a/b**, *O*-methylation, followed by keto-reduction to form **1** and **2** (Scheme 1). We set out to identify the missing oxidase that would hydroxylate cinchonidinone/cinchoninone (**15 a/b**). In *C. pubescens*, **1** and **2**, along with the C10–C11 dihydro analogs (**5** and **6**), accumulate only in roots and stem,^[8d]^ leading us to hypothesize that the **15 a/b**-hydroxylase would be specifically expressed in these two tissues. RNA-seq data from *C. pubescens* were therefore mined and a total of 150 putative oxidases (cytochrome P450s, flavin-containing monooxygenases, flavin adenine dinucleotide-dependent enzymes, and polyphenol oxidases) that were enriched in these tissues were selected, cloned into *Agrobacterium tumefaciens* and transiently expressed in *N. benthamiana* leaves. Infiltration of **15 a/b** into the treated *N. benthamiana* leaves, followed by liquid chromatography–mass spectrometry (LC–MS) analysis of the crude transformed leaf extracts, indicated that none of the enzyme candidates could hydroxylate or otherwise metabolize **15 a/b**.

This failure to identify **15 a/b**-hydroxylase, along with previously reported unsuccessful attempts,^[8d]^ prompted us to consider that methoxylation may occur at an earlier stage of the biosynthetic pathway. Strictosidine (**12**), dihydrocorynantheal (**18**), and cinchonamine (**19**) were tested as substrates with the oxidase gene candidates as described above, but no activity could be detected. Therefore, to pinpoint the stage at which methoxylation occurs, we performed untargeted and targeted metabolomics analyses of young tissue from *C. pubescens* leaves, stem, and root. These analyses revealed the presence of the putative intermediates tryptamine (**10**), strictosidine (**12**), corynantheal (**13**), dihydrocorynantheal (**18**), cinchoni(di)none (**15 a/b**) (along with cinchonamine (**19**) and tetrahydroalstonine (**20**), which are not predicted to be on pathway for **1** and **2**) (Figures S2–S7). Notably, for each of these metabolites a compound corresponding to the methoxylated analog was also detected (Figures S2–S7). These observations clearly indicated that two parallel pathways, one with methoxy and one with non-methoxy substrates, operate in *C. pubescens* (Scheme 2). The detection of 5-methoxytryptamine (**21**) in *C. pubescens* suggested that the aromatic hydroxylation occurs either on tryptamine (**10**) or tryptophan (**9**). However, the hydroxylated or methoxylated derivatives of tryptophan (**9**) were not detected in these metabolomic analyses.^[9]^ Therefore, we speculated that 5-methoxytryptamine (**21**) derives from hydroxylation of tryptamine (**10**) to form serotonin (**27**), which is then *O*-methylated to form **21** (Scheme 2).

To identify *C. pubescens* enzyme candidates that catalyze formation of 5-hydroxytryptamine (**27**) from tryptamine (**10**), we BLAST-searched for orthologs of CYP71P1 from *Oryza sativa*, the only reported functionally characterized tryptamine-5-hydroxylase.^[10]^ We transiently expressed the 15 top candidates in *N. benthamiana* leaves and infiltrated tryptamine substrate **10** into these infected leaves. LC–MS analysis of the resulting leaf extracts indicated the formation of **27** (*m/z* 177.102 [M+H]^+^) only in *N. benthamiana* leaves expressing either of two highly similar candidate enzymes (97.9% identical amino acids, Figure S8), which we thus named *Cp*T5H1 and *Cp*T5H2 (Figure 1a). Notably, *Cp*T5H1 and *Cp*T5H2 have complementary gene expression profiles (Figure 1b), consistent with the presence of different methoxylated alkaloid types (quinoline-, indole-, yohimbine-, and corynanthe-type) across different *C. pubescens* tissues (Figures S2–S7, and S9).

Subsequent *O*-methylation of serotonin (**27**) is required to install the methoxy group on 5-methoxytryptamine (**21**). To identify candidate enzymes that methylate the hydroxyl function of **27**, we searched for transcripts annotated as hydroxyindole-*O*-methyltransferase genes in the *C. pubescens* transcriptome, focusing on those that were expressed in all tissues (fragments per kilobase per million mapped fragments (FPKM) >4) and having a similar co-expression profile with either *Cp*T5H1 or *Cp*T5H2 (Pearson correlation coefficient >0.5). From this process, seven candidates were identified, expressed in *Escherichia coli*, purified, and assayed in vitro using **27** as a substrate. Among these candidates was the previously^[8d]^ identified 6’-hydroxycinchoninone-*O*-methyltransferase (*Cp*OMT1) mentioned above. Surprisingly, only *Cp*OMT1 showed methyltransferase activity with **27**, and the methylated product was identical to an authentic standard of 5-methoxytryptamine (**21**) (Figure 1a). This enzyme more efficiently consumed 5-hydroxytryptamine (**27**) than 6’-hydroxycinchoninone (**16 a/16b**) (substrate conversion after 60 min: >80% and <20%, respectively, Figure S10). This finding was corroborated by assays using transient expression in *N. benthamiana*, where **27** was almost fully converted to **21** after 3 h, while no methylation activity on 6’-hydroxycinchoninone (**16 a**/**b**) could be detected, even after 2d. Together, these data suggest that **27** is the native substrate for *Cp*OMT1. We further tested *in planta* the catalytic activity of *Cp*OMT1 on two other hydroxy-indole substrates, 5-hydroxytryptophan and *N*-acetyl-serotonin. We also tested the phenolic substrates quercetin-3-β-glucoside, quercitrin and caffeic acid, since *C. pubescens* also produces a wide range of phenolic natural products (Figure S11). Only *N*-acetyl-serotonin was methylated by *Cp*OMT1 to yield melatonin (Figure S12a), though since this compound was not detected in *C. pubescens*, this enzyme activity may not be physiologically relevant. Moreover, in substrate competition assays, *Cp*OMT1 preferred **27** over *N*-acetyl-serotonin (65% and 40% of conversion, respectively, Figure S12b). Finally, *Cp*OMT1 appears to be evolutionarily related to aromatic *O*-methyltransferases from other monoterpenoid indole alkaloid pathways (Figure S13a). We henceforth reclassify *Cp*OMT1 as a serotonin-*O*-methyltransferase.

We then characterized how *Cp*T5H1/2 and *Cp*OMT1 impact the ratio of methoxylated to non-methoxylated products when reconstituted with previously reported downstream *Cinchona* pathway enzymes *in planta*. Specifically, we intended to determine if downstream enzymes *Cp*DCS and *Cp*DCE, which produce intermediate dihydrocorynantheal (**18**) from strictosidine aglycone,^[8d]^ could produce both methoxy and non-methoxy products. However, while previous feeding studies have shown that many downstream monoterpene indole alkaloid pathway enzymes turn over substituted tryptamine analogs,^[11]^ known strictosidine synthase (STR) enzymes do not turn over tryptamine substrates with substituents at the C-5 position.^[12]^ Rationalizing that the *C. pubescens* strictosidine synthase must be able to turn over both tryptamine and 5-methoxytryptamine, we identified and functionally characterized strictosidine synthase from *C. pubescens*, (*Cp*STR) (Figure S14). We also identified two off-pathway enzymes (*Cp*THAS1 and *Cp*THAS2) that reduce the reactive strictosidine aglycone into tetrahydroalstonine **20** (Figure S15).

We transiently co-expressed *Cp*T5H, *Cp*OMT1, *Cp*STR, *Cp*DCS, *Cp*DCE, *Cp*THAS1, and *Catharanthus roseus* strictosidine glucosidase (*Cr*SGD, previously^[13]^ shown to accept 10-methoxy strictosidine) in *N. benthamiana* leaves along with co-infiltration of tryptamine (**10**) and secologanin (**11**). LC–MS analysis of the transformed leaf extracts showed the presence of 5-methoxytryptamine (**21**) in addition to the downstream products strictosidine (**7**), dihydrocorynantheal (**18**), tetrahydroalstonine (**20**), along with the respective methoxylated analogs (Figures 1c and S16). We also noticed that the aldehydes dihydrocorynantheal (**18**) and methoxy dihydrocorynantheal (**24**) were reduced into the corresponding alcohols **34** and **35** by endogenous tobacco reductases (Figure S17). While these results demonstrated that *Cp*STR and all downstream enzymes could turn over both methoxy and non-methoxy intermediates, we noted that all observed dihydrocorynantheal-type products (**18**, **24**, **34**, or **35**) were produced in low amounts (Figure 2d). In contrast, 10-methoxystrictosidine (**22**) and strictosidine (**12**) accumulated to higher levels (Figure 1d). We hypothesized that 10-methoxystrictosidine (**22**) and strictosidine (**12**), which are likely produced in the vacuole,^[14]^ are not efficiently exported from the vacuole of *N. benthamiana*, and thus cannot access the downstream cytosolic enzymes. Therefore, we searched for and identified an ortholog of a *Catharanthus roseus* strictosidine transporter^[15]^ in the *C. pubescens* transcriptome (*Cp*STTr), which, when co-expressed in *N. benthamiana* leaves along with other biosynthetic enzymes, improved the metabolic flux of both strictosidine (**12**) and 10-methoxystrictosidine (**22**), as evidenced by the relatively higher levels of downstream products, most notably 10-methoxydihydrocorynantheol (**35**) (Figure 1e).

Methoxylated alkaloids appear to be present in larger quantities than non-methoxy alkaloids in *Cinchona* mature plants.^[3a,16]^ We also observed substantial overproduction of methoxylated products compared to non-methoxy products in *N. benthamiana* reconstitution, though only when the transporter *Cp*STTr was included. The observed ratios of combined production of methoxylated aldehyde **24** and corresponding alcohol **35** versus combined formation of their non-methoxylated counterparts **18** and **34** were 1.23 and 0.23 with and without the transporter, respectively. This highlights how the inclusion of a vacuole transporter can impact the distribution of products, demonstrating the key role transporters can play in controlling product distribution both in the native producing organism and when heterologously reconstituting pathways. Moreover, these results indicate that *Cp*STR, *Cp*DCS, and *Cp*DCE are not specific for non-methoxylated substrates. This is particularly remarkable for *Cp*STR, since orthologs from *C. roseus* (*Cr*STR) and *Rauvolfia serpentina* (*Rs*STR) are highly specific for tryptamine.^[17]^ We separately tested *Cp*STR in *N. benthamiana* with tryptamine (**10**), 5-methoxytryptamine (**21**) and 5-hydroxytryptamine (**27**) confirming that this enzyme turns over all of these substrates (Figures S18 and S19). This is consistent with early studies using enzyme activity purified from *Cinchona robusta* suspension culture.^[18]^ Notably, it has been shown that a single point mutation enables *Cr*STR to accept C5-substituted tryptamine analogs,^[12]^ but this mutation is not observed in *Cp*STR. Instead, we noted that *Cp*STR is phylogenetically distant to *Cr*STR and *Rs*STR (Figure S20), which are part of alkaloid pathways where the aromatic methoxylation occurs at a late biosynthetic stage, after formation of strictosidine.

We next wanted to conclusively demonstrate that hydroxylation at this early biosynthetic stage actually occurs in *C. pubescens*, particularly since *Cp*OMT1 can also methylate 6’-hydroxycinchoninone (**16a/16b**), albeit at low efficiency. Therefore, we synthesized isotopically labeled tryptamine-(*indole*-*d*5) (**10a**) and 5-methoxytryptamine-(O-*methyl*-^*13*^*C*, *d*3) (**21a**) and administrated these two compounds separately to roots and leaf tissues from in vitro generated *C. pubescens* plantlets (Figure 2a). Gratifyingly, both labeled substrates were incorporated into quinine (**1**) and quinidine (**2**) (Figure 2b) along with other related alkaloids (Figures S21–S29), as evidenced by LC–MS analysis. In particular, the incorporation of 5-methoxytryptamine (**21**) into **1** and **2**, along with the detection of 5-methoxytryptamine-(*indole*-*d*4) **21b** in the feeding experiments with labeled tryptamine (**10a**) (Figure S25), firmly demonstrated that **1** and related methoxylated metabolites derive from 5-methoxytryptamine (**21**), which, in turn, is formed from tryptamine (**10**) (Schemes 2 and S1).

Since the non-methoxylated alkaloid corynantheal (**13**) had been suggested to be an intermediate of the methoxylated metabolites **1** and **2**,^[8b]^ we also wanted to conclusively determine whether **13** would be incorporated using our established system for feeding studies. Since labeled **13** was not available, we enzymatically synthesized an aromatic ring-*d*4 labeled form of the dihydro analog **18** (i.e., **18a**, Figure S30), to track its incorporation into the downstream dihydro quinine-like compounds present in *Cinchona*. Feeding tissues of *Cinchona* plantlets with **18a** followed by LC–MS analyses of the resulting extracts revealed that **18a** was incorporated into dihydrocinchonidine and dihydrocinchonine (**7** and **8**) (Figure S30). Importantly however, there was no observable conversion of **18a** into the dihydro methoxylated analogs **5** and **6**. Moreover, the labeled isotopic pattern of **18a** was observed in a metabolite tentatively assigned as dihydrocinchonamine **32** (a mid-pathway biosynthetic shunt alkaloid, Figure S31). Hence, this experiment corroborated the finding that methoxylation occurs prior to the formation of **18** ((dihydro)corynantheal) and **32** ((dihydro)cinchonaminal)). In addition, these feeding studies also suggest that the biogenic relationship between **1**–**4** and the respective dihydro analogs **5**–**8** diverge early in the pathway, most likely by equilibration of the cyclized forms of strictosidine aglycone as previously^[8d]^ proposed.

In conclusion, metabolomics, transcriptomics and feeding experiments using stable isotope-labeled precursors demonstrate that the biosynthesis of quinine (**1**) and related methoxylated Cinchona alkaloids are initiated by the hydroxylation of tryptamine, followed by *O*-methylation to produce 5-methoxytryptamine. The co-occurrence of both tryptamine and 5-methoxytryptamine *in planta* and the promiscuity of the pathway enzymes drive concomitant parallel formation of methoxylated and non-methoxylated alkaloids in *C. pubescens* by the same pathway enzymes. We additionally showed that we could modulate the product profile by inclusion of a vacuolar transporter in reconstitution experiments. This work therefore sets the stage for production of valuable Cinchona alkaloids via synthetic biology.

## Supplementary Material

1

The Supporting Information contains detailed methods and materials. Likewise contained are supplementary scheme, figures and data including chemical structures, LC–MS results, gene nucleotide sequences, phylogenetic analysis, and characterization data of synthesized labeled compounds.

The authors have cited additional references within the Supporting Information.^[19–36]^

## Figures and Tables

**Figure 1.**
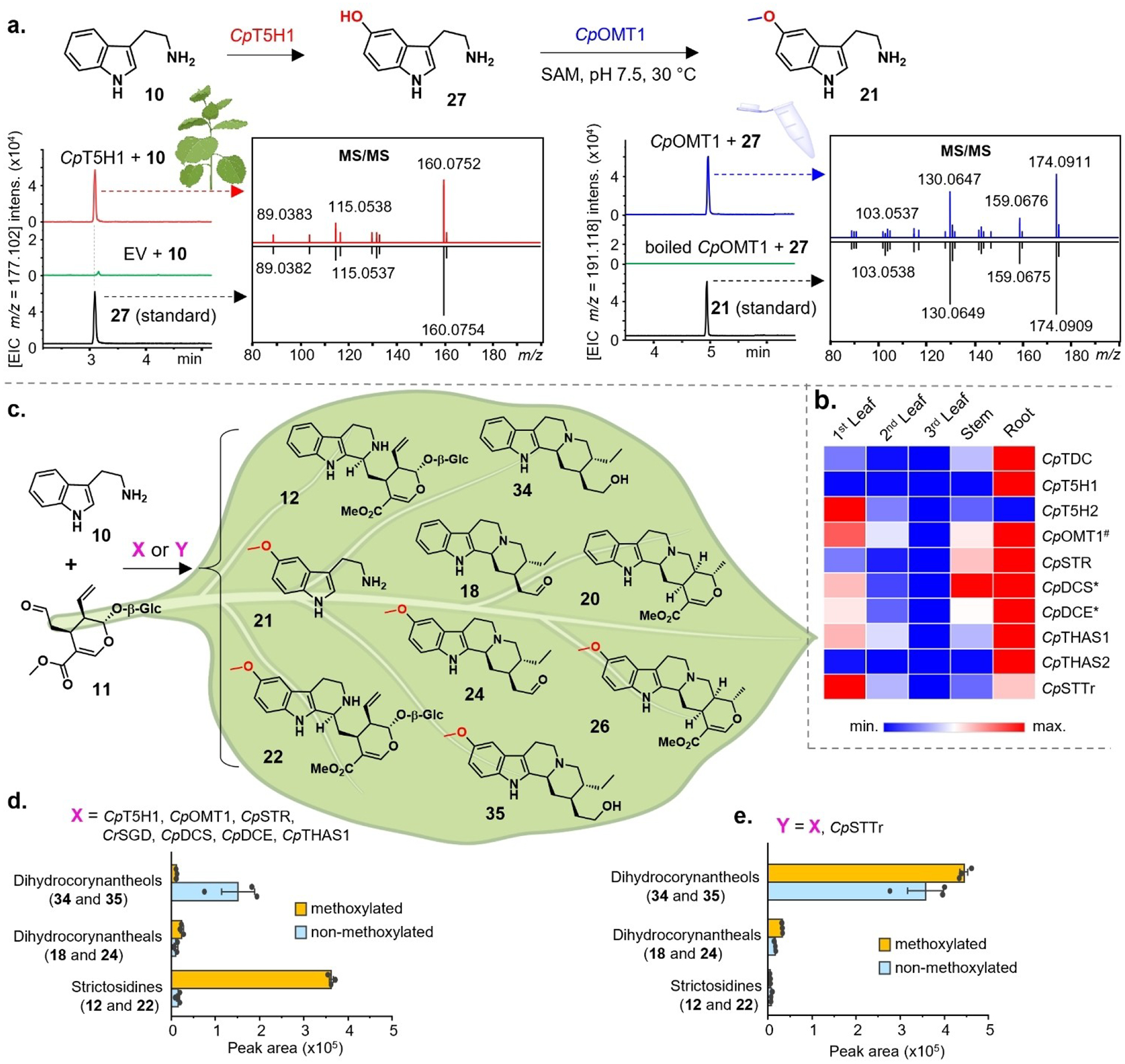
**(a)** Identification and functional characterization of 5-methoxytryptamine (**21**) biosynthetic genes; **(b)** expression profiles of identified genes in *C. pubescens* (* indicates previously reported genes; # denotes previously reported but here functionally repurposed gene); **(c)** reconstruction of the pathways to early Cinchona alkaloids in *N. benthamiana* (for further details see Figure S16); **(d)** LC–MS peak areas of key metabolites, strictosidines and corynantheal-type alkaloids, produced when only biosynthetic genes are co-expressed in *N. benthamiana* leaves (Date are mean ± s.e.m.; *n*=3 biological replicates); and **(e)** accumulation of the same key metabolites when the transporter gene *Cp*STTr is co-expressed with the biosynthetic genes in *N. benthamiana* leaves (Date are mean ± s.e.m.; *n*=3 biological replicates). The plant image indicates that the displayed data are from *in planta* biochemical assays while the image of the tube indicates that the data are from in vitro biochemical assays.

**Figure 2.**
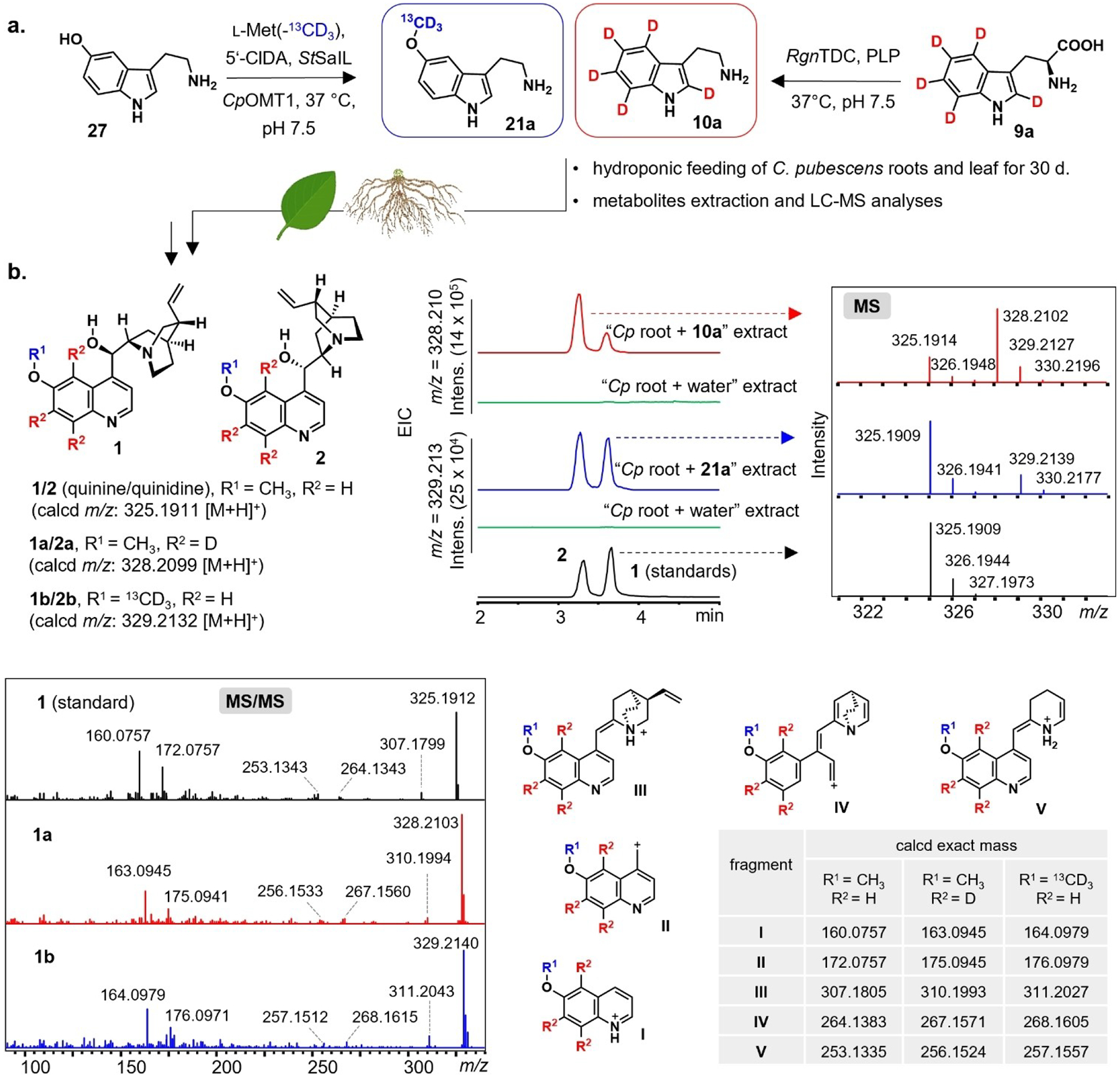
**(a)** synthesis of the labeled precursors tryptamine-(*indole*-*d*5) (**10a**) and 5-methoxytryptamine-(O-*methyl*-^13^C, *d*3) (**21a**), which were separately fed to the roots and leaves of *C. pubescens*; **(b)** key methoxylated Cinchona alkaloids **1** and **2**, and extracted ion chromatograms of the corresponding isotope-labeled analogs, MS isotopic patterns, MS/MS spectrum (20.0–50.0 eV), and selected putative MS/MS fragments **I–V**, all evidencing the incorporation of **21a** and **10a**.

**Scheme 1.**
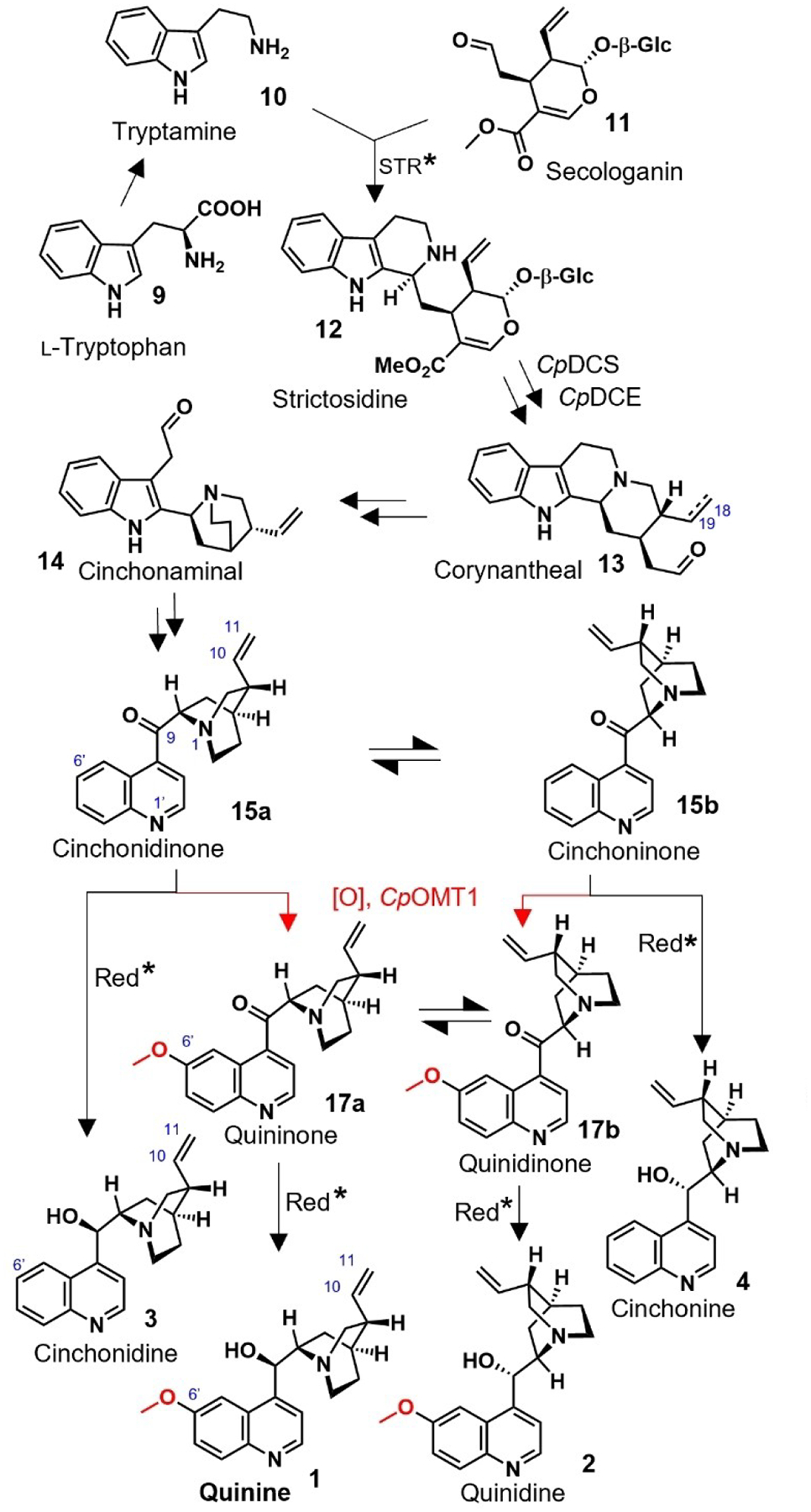
Previously accepted hypothetical biosynthetic pathway to quinine (**1**) and congeners **2**–**4**. Highlighted in red are the putative steps installing the methoxy group. The asterisk designates previously reported proteins that have been biochemically characterized from crude *Cinchona* tissues, but the corresponding gene remains undiscovered. The dashed double bond at C18–C19 in **13** indicates that this compound also occurs in the dihydro form (i.e., with a single bond, see **18**).

**Scheme 2.**
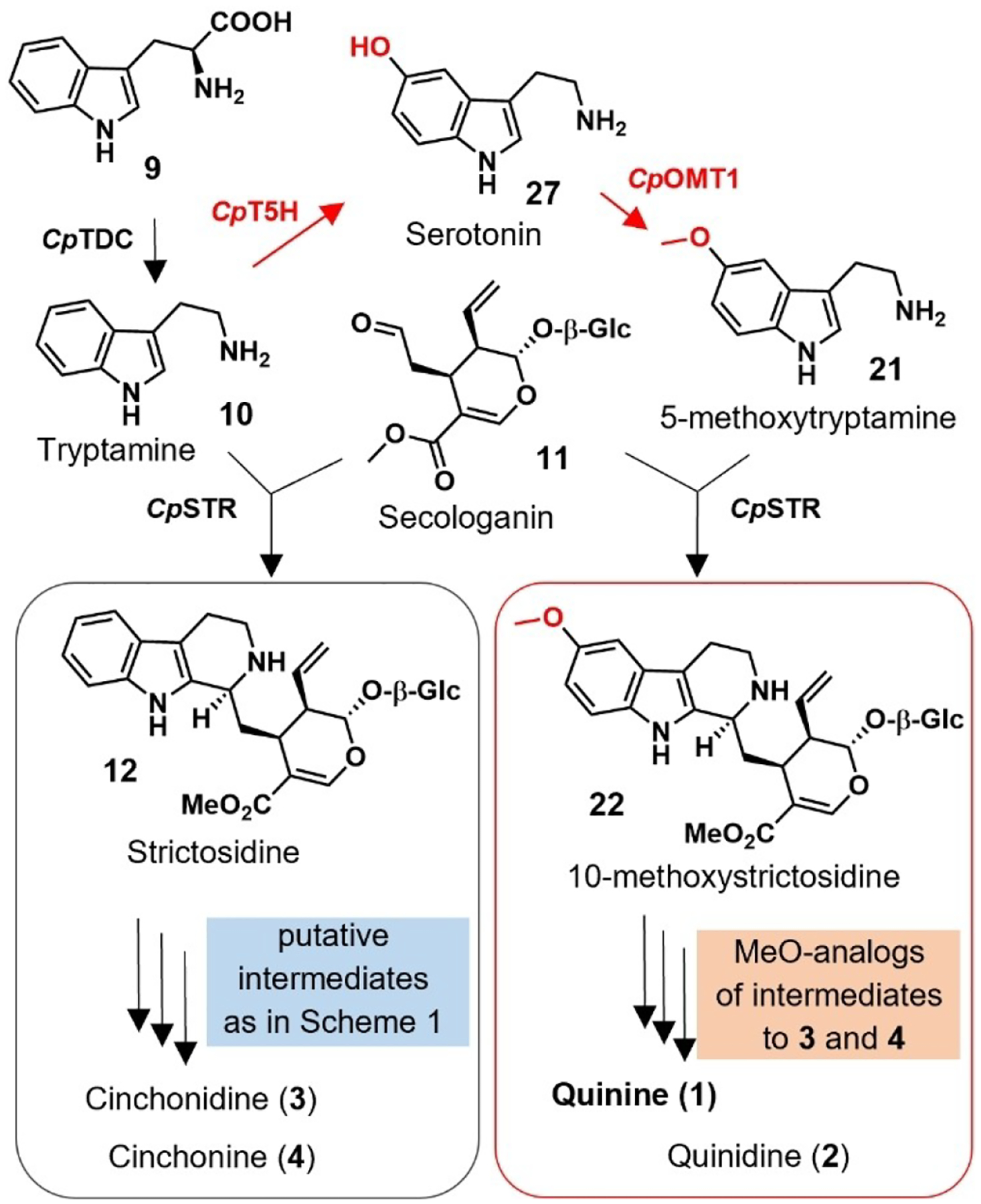
Revised biosynthetic pathway for Cinchona alkaloids. The key steps and involved enzymes that introduce the aromatic methoxy group and leading to parallel pathways are highlighted in red. For a detailed scheme see Scheme S1.

## Data Availability

The data that support the findings of this study are available in the supplementary material of this article.

## References

[1] a)Achan J, Talisuna AO, Erhart A, Yeka A, Tibenderana JK, Baliraine FN, Rosenthal PJ, D’Alessandro U, Malar. J 2011, 10, 144; 21609473 10.1186/1475-2875-10-144 PMC3121651

[2] a)Grace AA, Camm AJ, New Eng. J. Med 1998, 338, 35–45; 9414330 10.1056/NEJM199801013380107

[3] a)Verpoorte R, Schripsema J, van der Leer T, in The Alkaloids: Chemistry and Pharmacology, Vol. 34 (Ed.: Brossi A), Academic Press, 1989, pp. 331–398;

[4] a)Marcelli T, van Maarseveen JH, Hiemstra H, Angew. Chem. Int. Ed 2006, 45, 7496–7504; 10.1002/anie.200602318 17051626

[5] a)Woodward RB, Doering WE, J. Am. Chem. Soc 1944, 66, 849–849;

[6] a)Leete E, Wemple JN, J. Am. Chem. Soc 1969, 91, 2698–2702; 5784947 10.1021/ja01038a049

[7] O’Connor SE, Maresh JJ, Nat. Prod. Rep 2006, 23, 532–547. 16874388 10.1039/b512615k

[8] a)Leete E, Acc. Chem. Res 1969, 2, 59–64;

[9] Murch SJ, KrishnaRaj S, Saxena PK, Plant Cell Rep. 2000, 19, 698–704. 30754808 10.1007/s002990000206

[10] Fujiwara T, Maisonneuve S, Isshiki M, Mizutani M, Chen L, Wong HL, Kawasaki T, Shimamoto K, J. Biol. Chem 2010, 285, 11308–11313. 20150424 10.1074/jbc.M109.091371 PMC2857009

[11] a)McCoy E, O’Connor SE, J. Am. Chem. Soc 2006, 128, 14276–14277; 17076499 10.1021/ja066787w

[12] Bernhardt P, McCoy E, O’Connor SE, Chem. Biol 2007, 14, 888–897. 17719488 10.1016/j.chembiol.2007.07.008 PMC2700010

[13] Yerkes N, Wu JX, McCoy E, Galan MC, Chen S, O’Connor SE, Bioorg. Med. Chem. Lett 2008, 18, 3095–3098. 18061449 10.1016/j.bmcl.2007.11.063 PMC2474460

[14] Courdavault V, Papon N, Clastre M, Giglioli-Guivarc’h N, St-Pierre B, Burlat V, Curr. Opin. Plant Biol 2014, 19, 43–50. 24727073 10.1016/j.pbi.2014.03.010

[15] Payne RME, Xu D, Foureau E, Teto Carqueijeiro MIS, Oudin A, Bernonville T. D. d., Novak V, Burow M, Olsen C-E, Jones DM, Tatsis EC, Pendle A, Ann Halkier B, Geu-Flores F, Courdavault V, Nour-Eldin HH, O’Connor SE, Nat. Plants 2017, 3, 16208. 28085153 10.1038/nplants.2016.208 PMC5238941

[16] a)Aerts RJ, de Waal A, Pennings EJM, Verpoorte R, Planta 1991, 183, 536–541; 24193847 10.1007/BF00194275

[17] a)McCoy E, Galan MC, O’Connor SE, Bioorg. Med. Chem. Lett 2006, 16, 2475–2478; 16481164 10.1016/j.bmcl.2006.01.098

[18] Stevens LH, Giroud C, Pennings EJM, Verpoorte R, Phytochemistry 1993, 33, 99–106.

[19] Boccia M, Grzech D, Lopes AA, O’Connor SE, Caputi L, Front. Plant Sci 2022, 13. 10.3389/fpls.2022.919443 PMC9257203 35812900

[20] Cárdenas PD, Sonawane PD, Heinig U, Jozwiak A, Panda S, Abebie B, Kazachkova Y, Pliner M, Unger T, Wolf D, Ofner I, Vilaprinyo E, Meir S, Davydov O, Gal-on A, Burdman S, Giri A, Zamir D, Scherf T, Szymanski J, Rogachev I, Aharoni A, Nat. Commun 2019, 10, 5169. 31727889 10.1038/s41467-019-13211-4 PMC6856131

[21] Berrow NS, Alderton D, Sainsbury S, Nettleship J, Assenberg R, Rahman N, Stuart DI, Owens RJ, Nucleic Acids Res. 2007, 35, e45–e45. 17317681 10.1093/nar/gkm047 PMC1874605

[22] Sparkes IA, Runions J, Kearns A, Hawes C, Nat. Protoc 2006, 1, 2019–2025. 17487191 10.1038/nprot.2006.286

[23] Schmid R, Heuckeroth S, Korf A, Smirnov A, Myers O, Dyrlund TS, Bushuiev R, Murray KJ, Hoffmann N, Lu M, Sarvepalli A, Zhang Z, Fleischauer M, Dührkop K, Wesner M, Hoogstra SJ, Rudt E, Mokshyna O, Brungs C, Ponomarov K, Mutabdžija L, Damiani T, Pudney CJ, Earll M, Helmer PO, Fallon TR, Schulze T, Rivas-Ubach A, Bilbao A, Richter H, Nothias L-F, Wang M, Orešič M, Weng J-K, Böcker S, Jeibmann A, Hayen H, Karst U, Dorrestein PC, Petras D, Du X, Pluskal T, Nat. Biotechnol 2023, 41, 447–449. 36859716 10.1038/s41587-023-01690-2 PMC10496610

[24] Dührkop K, Fleischauer M, Ludwig M, Aksenov AA, Melnik AV, Meusel M, Dorrestein PC, Rousu J, Böcker S, Nat. Methods 2019, 16, 299–302. 30886413 10.1038/s41592-019-0344-8

[25] Dührkop K, Nothias L-F, Fleischauer M, Reher R, Ludwig M, Hoffmann MA, Petras D, Gerwick WH, Rousu J, Dorrestein PC, Böcker S, Nat. Biotechnol 2021, 39, 462–471. 33230292 10.1038/s41587-020-0740-8

[26] Kim HW, Wang M, Leber CA, Nothias L-F, Reher R, Kang KB, van der Hooft JJJ, Dorrestein PC, Gerwick WH, Cottrell GW, J. Nat. Prod 2021, 84, 2795–2807. 34662515 10.1021/acs.jnatprod.1c00399 PMC8631337

[27] Wang M, Carver JJ, Phelan VV, Sanchez LM, Garg N, Peng Y, Nguyen DD, Watrous J, Kapono CA, Luzzatto-Knaan T, Porto C, Bouslimani A, Melnik AV, Meehan MJ, Liu W-T, Crüsemann M, Boudreau PD, Esquenazi E, Sandoval-Calderón M, Kersten RD, Pace LA, Quinn RA, Duncan KR, Hsu C-C, Floros DJ, Gavilan RG, Kleigrewe K, Northen T, Dutton RJ, Parrot D, Carlson EE, Aigle B, Michelsen CF, Jelsbak L, Sohlenkamp C, Pevzner P, Edlund A, McLean J, Piel J, Murphy BT, Gerwick L, Liaw C-C, Yang Y-L, Humpf H-U, Maansson M, Keyzers RA, Sims AC, Johnson AR, Sidebottom AM, Sedio BE, Klitgaard A, Larson CB, Boya CA, Torres-Mendoza PD, Gonzalez DJ, Silva DB, Marques LM, Demarque DP, Pociute E, O’Neill EC, Briand E, Helfrich EJN, Granatosky EA, Glukhov E, Ryffel F, Houson H, Mohimani H, Kharbush JJ, Zeng Y, Vorholt JA, Kurita KL, Charusanti P, McPhail KL, Nielsen KF, Vuong L, Elfeki M, Traxler MF, Engene N, Koyama N, Vining OB, Baric R, Silva RR, Mascuch SJ, Tomasi S, Jenkins S, Macherla V, Hoffman T, Agarwal V, Williams PG, Dai J, Neupane R, Gurr J, Rodríguez AMC, Lamsa A, Zhang C, Dorrestein K, Duggan BM, Almaliti J, Allard P-M, Phapale P, , Nat. Biotechnol 2016, 34, 828–837. 27504778 10.1038/nbt.3597 PMC5321674

[28] Nothias L-F, Petras D, Schmid R, Dührkop K, Rainer J, Sarvepalli A, Protsyuk I, Ernst M, Tsugawa H, Fleischauer M, Aicheler F, Aksenov AA, Alka O, Allard P-M, Barsch A, Cachet X, Caraballo-Rodriguez AM, Da Silva RR, Dang T, Garg N, Gauglitz JM, Gurevich A, Isaac G, Jarmusch AK, Kameník Z, Kang KB, Kessler N, Koester I, Korf A, Le Gouellec A, Ludwig M, Martin H C, McCall L-I, McSayles J, Meyer SW, Mohimani H, Morsy M, Moyne O, Neumann S, Neuweger H, Nguyen NH, Nothias-Esposito M, Paolini J, Phelan VV, Pluskal T, Quinn RA, Rogers S, Shrestha B, Tripathi A, van der Hooft JJJ, Vargas F, Weldon KC, Witting M, Yang H, Zhang Z, Zubeil F, Kohlbacher O, Böcker S, Alexandrov T, Bandeira N, Wang M, Dorrestein PC, Nat. Methods 2020, 17, 905–908. 32839597 10.1038/s41592-020-0933-6 PMC7885687

[29] McDonald AD, Perkins LJ, Buller AR, ChemBioChem 2019, 20, 1939–1944. 30864270 10.1002/cbic.201900069 PMC6800669

[30] Eustáquio AS, Pojer F, Noel JP, Moore BS, Nat. Chem. Biol 2008, 4, 69–74. 18059261 10.1038/nchembio.2007.56 PMC2762381

[31] Tamura K, Stecher G, Kumar S, Mol. Biol. Evol 2021, 38, 3022–3027. 33892491 10.1093/molbev/msab120 PMC8233496

[32] Zuckerkandl E, Pauling L, in Evolving Genes and Proteins (Eds.: Bryson V, Vogel HJ), Academic Press, 1965, pp. 97–166.

[33] Fujiwara T, Maisonneuve S, Isshiki M, Mizutani M, Chen L, Wong HL, Kawasaki T, Shimamoto K, J. Biol. Chem 2010, 285, 11308–11313. 20150424 10.1074/jbc.M109.091371 PMC2857009

[34] Edgar RC, Nucleic Acids Res. 2004, 32, 1792–1797. 15034147 10.1093/nar/gkh340 PMC390337

[35] Robert X, Gouet P, Nucleic Acids Res. 2014, 42, W320–W324. 24753421 10.1093/nar/gku316 PMC4086106

[36] Letunic I, Khedkar S, Bork P, Nucleic Acids Res. 2021, 49, D458–D460. 33104802 10.1093/nar/gkaa937 PMC7778883

